# Clinical Outcomes and Prognostic Factors of Patients With Early Malignant Melanoma in One Latin American Country: Results of the Epidemiological Registry of Malignant Melanoma in Colombia Study

**DOI:** 10.1200/GO.22.00377

**Published:** 2023-05-22

**Authors:** Aylen Vanessa Ospina Serrano, Fernando Contreras, Iván Triana, Guillermo Sánchez-Vanegas, Juan David Ortíz, Pedro Ramos, Claudia Vargas, Natalia Arango, Henry Idrobo, Isabel Munévar, Andrés Yepes, William Mantilla, Paola Jiménez, Giovanna Rivas, Mauricio Lema, Carmen Alcalá, Diego Gómez, Matilde Chinchia, Angela Barrero

**Affiliations:** ^1^ Asociación Colombiana de Hematología y Oncología—ACHO, ICCAL Hospital, Universitario Fundación Santa Fe de Bogotá, Bogotá, Colombia; ^2^ Instituto Nacional de Cancerología INC, Universidad El Bosque, Bogotá, Colombia; ^3^ Hospital Universitario Mayor—Mederi, Universidad del Rosario, Bogotá, Colombia; ^4^ Fundación Valle de Lili, Cali, Colombia; ^5^ Clínica Universitaria Colombia Sanitas, Oncocare, Bogotá, Colombia; ^6^ Centro Oncológico Antioquia, Medellín, Colombia; ^7^ Centro Médico Julián Coronel, Universidad del Valle, Cali, Colombia; ^8^ Hospital Militar Central, Fundación Cardio infantil, Hemato Oncólogos Asociados, Bogotá, Colombia; ^9^ Hospital Universitario San Vicente Fundación, Medellín, Colombia; ^10^ Fundación Cardio infantil, Universidad del Rosario, Grupo ICAROS, Bogotá, Colombia; ^11^ Hemato Oncólogos Asociados, Los Cobos Medical Center, Bogotá, Colombia; ^12^ Clínica de Occidente, Cali, Colombia; ^13^ Clínica de Oncología Astorga, Medellín, Colombia; ^14^ Clínica de la Costa, Barranquilla, Colombia; ^15^ Fundación cardiovascular de Colombia, Hospital Internacional de Colombia, Bucaramanga, Colombia; ^16^ Cosmitet, Clínica Rey David, Cali, Colombia; ^17^ Asociación Colombiana de Hematología y Oncología—ACHO, Bogota, Colombia

## Abstract

To describe the population with early malignant melanoma, we performed a cohort study on the basis of the Epidemiological Registry of Malignant Melanoma in Colombia-Asociacion Colombiana de Hematologia y Oncologia. From January 2011 until December 2021, 759 patients were included; the average age was 66 years, 57% were women, acral lentiginous histology was found in 27.8% of patients, and the median follow-up was 36.5 months. The prognostic factors for overall survival in our population are Eastern Cooperative Oncology Group 3-4 (hazard ratio [HR], 13.8), stage III (HR, 5.07), received radiotherapy (HR, 3.38), ulceration on histology (HR, 2.68), chronic sun exposure (HR, 2.3), low income (HR, 2.04), previous local surgery (HR, 0.27), and have received adjuvant treatment (HR, 0.41).

## INTRODUCTION

According to the global burden of skin cancer disease study, melanoma has significantly increased during past years, with the prevalence from 20 cases per 100,000 people in 1990 to 30 cases per 100,000 people in 2017.^1^ In the context of Latin American countries, such as Colombia, despite being considered an uncommon disease, progressive frequency has been documented, with a reported incidence of six new cases per 100,000 persons in 2007 and with an estimated projection of 14 new cases per 100,000 persons in 2020.^2^

**CONTEXT**

**Key Objective**
Are the characteristics of patients with early melanoma in Colombia different from other populations?
**Knowledge Generated**
There are similar factors to those described worldwide for recurrence, survival, and protectives in our population. However, low income is an important additional poor prognosis determinant. The most frequent histology was acral lentiginous; however, it could not be associated with poor prognosis.
Overall, patients have lower 5-year survival than North American population (83% versus 99.4%). This may be due to higher diagnosis in locally advanced stages as consequence of delays in access to medical attention.
**Relevance**
Local information on the characteristics of patients with early melanoma has been limited. Our findings offer support for clinical decision making and to generate specific strategies for Colombian patients. Despite the difficulties, it is important to continue with this research and to have longer follow-up times, to achieve more information and greater precision of the collected data.

Given the lethality of the neoplasm, it has been a permanent interest to establish prognostic factors in patients with melanoma and to apply strategies for modifying the clinical course of the disease and increasing overall survival (OS).^3-5^ The risk factors usually described are age, male sex, presence of ulceration on histology, tumor thickness, histological type, treatment received, and tumor staging.^3-5^ However, there is a lack of information regarding the behavior of this neoplasia in Latin America, specifically in the Colombian population, which features a process of miscegenation and specific sociodemographic conditions, which could determine a set of prognostic factors different from those historically described.

Regarding the approach to high-risk melanoma with locoregional involvement, important advances have been made recently, such as the administration of adjuvant treatment with immune checkpoint inhibitors or anti-BRAF/anti–MEK therapy,^6-11^ as well as the reassessment of the therapeutic value of lymphadenectomy.^12-14^ These strategies have improved patient survival and reduced surgical treatment morbidity.

Given the lack of consolidated local data, the Asociacion Colombiana de Hematologia y Oncologia (ACHO) developed the Epidemiological Registry of Malignant Melanoma in Colombia (REMMEC), intending to characterize the population and establish clinical outcomes and related prognostic factors in patients with early malignant melanoma in Colombia.

## MATERIALS AND METHODS

### Design, Population, and Sample

An analytical observational cohort study was carried out on the basis of the REMMEC registry, which was generated by the ACHO. Researchers from 16 health institutions of the country belonging to the national health system, none of them private, participated in this study.

Recruitment was performed from January 2011 to December 2021. Inclusion criteria were age older than 18 years and malignant melanoma histologically confirmed and clinical stage 0-III according to 8th edition American Joint Committee on Cancer system.

The sample size to determine prognostic factors was based on the method described by Lachin et al.^22^ The assumptions for the calculation were based on the estimates reported in the literature.^9,12^ The outcome of 5-year mortality was used for the variables of high-medium socioeconomic level compared with low (58% *v* 75%), presence of ulceration (9% *v* 38%), and Breslow <0.8 (5% *v* 15%). To obtain a power of 80% and a significance of 95%, a minimum sample size of 680 patients was calculated.

### Enrollment, Monitoring, and Data Processing Procedure

The REMMEC registry involved a group of medical oncologists and hematologist oncologists who responded to the call made by the ACHO. Patients who met the selection criteria were incorporated into the data capture system. Written informed consent was provided by participants. The researchers from each center had a personal username and password to enter information into a data collection questionnaire created for the project that was hosted on a virtual platform. The point of admission to the cohort was determined by the confirmed diagnosis of localized or locally advanced cutaneous malignant melanoma. Since their admission to the cohort, these patients have had at least a quarterly follow-up period.

### Variables of Interest

The primary outcome was OS, calculated on the basis of the follow-up time between admission to the cohort and the date of the event (all-cause mortality) or censoring. The secondary outcome was defined as tumor recurrence-free survival (RFS) on the basis of the time from the initial diagnosis to the time of tumor recurrence or the detection of locoregional or distant metastases after oncological treatment of the primary tumor.

Possible prognostic factors such as age, sex, phototype, history of chronic sun exposure (constant and prolonged exposure to ultraviolet radiation [UVR] related to permanent or routine recreational or work activities), Eastern Cooperative Oncology Group index (ECOG—Performance Status) on admission, location, histological type, TNM staging, Breslow classification, ulceration, and treatment received were analyzed. In addition, we included social factors such as socioeconomic level and type of health insurance (contributory or subsidized) as indicators of access to receive opportune health care. This, because it is known that in low- and middle-income countries such as ours, low socioeconomic level is a factor that obstructs access to the health care system. In Colombia, the socioeconomic or income level is defined through categories on the basis of the structural facilities of the housing and the neighborhood in which people live. The levels range from I (lowest level) to VI (highest level) and the Colombian health system is divided into two regimens: contributory and subsidized. People who belong to the contributory system are those who have the capacity to pay and provide resources to the health system. People without the capacity to pay, who generally belong to the lower social level, belong to the subsidized health regimen and receive solidarity benefits from the health system.

### Statistical Analysis

A descriptive analysis was carried out according to the measurement scale of the different variables. Absolute and relative frequency measures were used for qualitative variables while numerical variables were described using measures of central tendency (mean and median) and dispersion (standard deviation and interquartile range).

Recurrence rate (recurrence events/sum person follow-up time) and case fatality rate (patients who died/sum person follow-up time) were estimated. OS and RFS were established using the Kaplan-Meier method. A multivariate analysis was performed using the Cox proportional hazards method to control for confounding factors, detect possible interactions, and estimate prognostic factors. Estimates with *P* values < .05 were considered statistically significant differences. The clinical relevance of the different factors and the verification of the proportionality assumption were considered in selecting the best model. The effect estimate was reported using the hazard ratio (HR) (95% CI). All analyses were performed with the Stata 17 statistical software (StataCorp, LLC, College Station, TX).

### Ethical Aspects

This study respected the ethical principles of the Declaration of Helsinki (2013) and the Resolution 8430 of 1993 of the Colombian Ministry of Health. The information was guaranteed to be for scientific purposes only, and privacy was protected by the omission of the identification data. The protocol was presented and approved by the ethics committees of all participating institutions.

## RESULTS

### Clinical and Sociodemographic Characteristics

A total of 759 patients were included, of whom 57% (431) were women. The average age was 66 years, with 50% of patients between 56 and 75 years. According to socioeconomic and income scale, 31.9% (242) were in low level and 5.2% (18) lived in rural areas. Regarding the type of health regimen, 74% (560) were in the contributory regimen. The patients that had no personal or family history of melanoma were 94.5% (717); 29% (219) were phototype I or II, and the most frequent histological type was acral lentiginous melanoma (27.8%, 211), followed by superficial spreading melanoma (18.2%, 138). The two most frequent locations were lower extremities (38%, 288) and face (25%, 189). Eighty-eight percent (670) were skin-originated. In the Breslow classification, 17% (130) were tumors smaller than 0.8 mm or in situ, and 34% (260) had ulceration. Regarding the stage of the disease, 13.9% (106) had stage 0, 24.2% (184) had stage I, 30% (228) had stage II, and 31.7% (241) had stage III.

Ninety-two percent (699) of patients underwent local surgery, and 30% (226) underwent local lymphadenectomy. The patients that received adjuvant treatment were 23.8% (181), of whom seven received targeted therapy, 82 interferon, and 92 immune checkpoints inhibitors. The median follow-up time was 36.5 months (IQR, 17.2-71.5 months). Tables 1 and 2 present general characteristics and findings in patients who presented recurrence or died during follow-up.

**TABLE 1:**
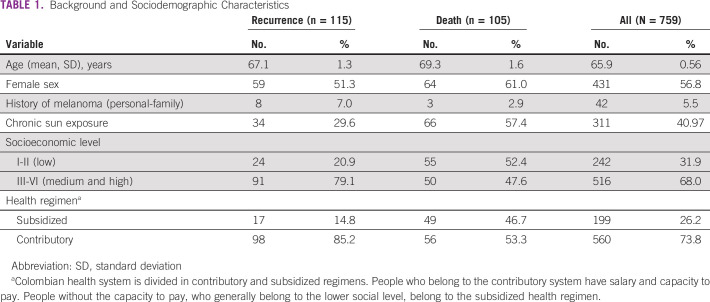
Background and Sociodemographic Characteristics

**TABLE 2:**
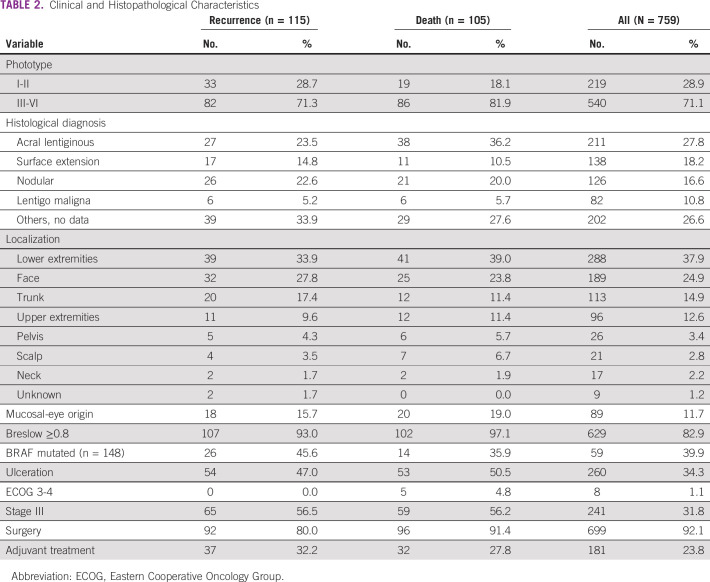
Clinical and Histopathological Characteristics

### Tumor Recurrence-Free Survival

A total of 115 recurrences were documented, contributing a total of 2,938 person-years, which translated into a recurrence rate of 3.9 patients per 100 person-years (95% CI, 3.2 to 4.6 per 100 person-years). According to the Kaplan-Meier function, the probability of RFS at 1 year was 97.2% (95% CI, 95.7 to 98.2); for the fifth year, it was 81.5% (95% CI, 77.6 to 84.8); and by the tenth year, it reached 67.8% (95% CI, 59.8 to 75.5).

### Prognostic Factors for RFS

The median age of the patients with recurrence was 67 years. The recurrence rate was 17% among men (56 of 328), compared with 14% among women (59 of 431), with no significant differences between groups documented (*P* = .19). The recurrence incidence rate is provided in Table 3. Factors that increase the probability of recurrence are ECOG 3-4 (HR, 9.71), stage III (HR, 4.65), presence of ulceration (HR, 2.5), belonging to subsidized regimen of the health system (HR, 2.19), and chronic sun exposure (HR, 2.07). On the other hand, the factors that protect against recurrence are adjuvant treatment (HR, 0.42), local surgery (HR, 0.25), and Breslow <0.8 (HR, 0.28); Table 4.

**TABLE 3:**
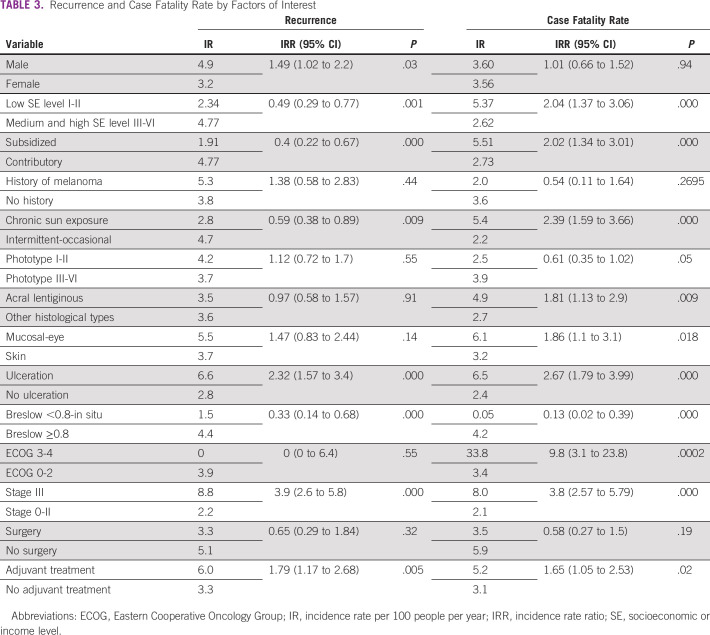
Recurrence and Case Fatality Rate by Factors of Interest

**TABLE 4:**
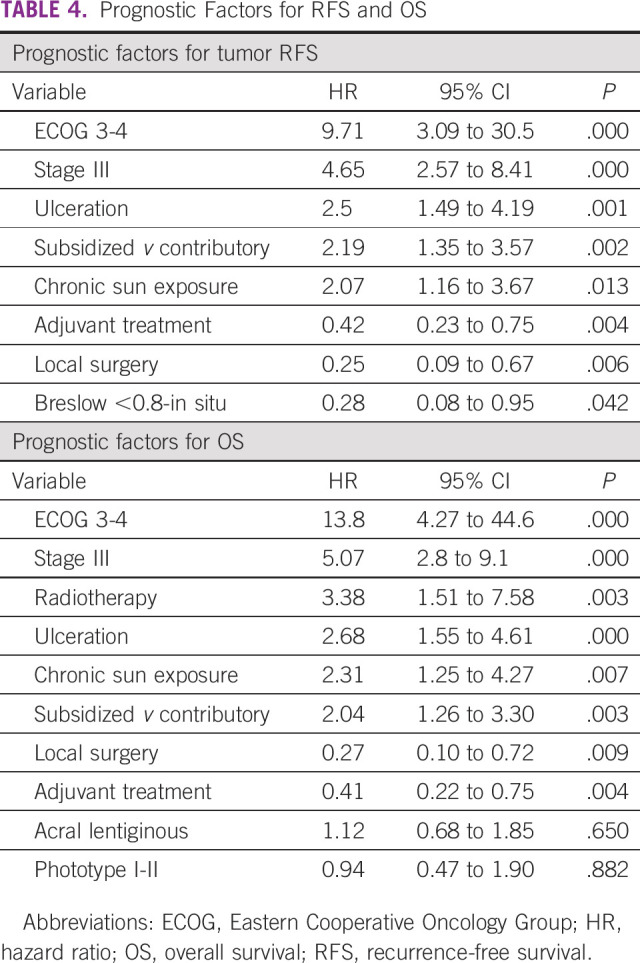
Prognostic Factors for RFS and OS

### Case Fatality Rate and Overall Survival

During the study, 13.8% (105) of patients died. The cumulative person time of this cohort was 2,938 person-years, for a case fatality rate of 3.6 cases per 100 person-years (95% CI, 2.9 to 4.3 per 100 person-years). The case fatality rate is provided in Table 3. According to the Kaplan-Meier function, OS after 1-year follow-up was 97.4% (95% CI, 95.9 to 98.3); for the fifth year, it was 83.2% (95% CI, 79.5 to 86.2); and for the tenth year, it reached 71.1% (95% CI, 63.8 to 77.3).

### Prognostic Factors for OS

In the group of patients who died, the median age was 71 years, compared with those who did not die (65 years, *P* = .006). Five-year OS for patients in stage 0 and I was 97% (95% CI, 89 to 99), stage II was 80% (95% CI, 72.6 to 86.6), and stage III was 65% (95% CI, 57 to 72). The case fatality distribution was 12.5% for men (41 of 328) and 14.8% for women (64 of 431). According to the multivariate analysis, the prognostic factors associated with lower OS were ECOG 3-4 (HR, 13.8), stage III (HR, 5.07), treatment with radiotherapy (HR, 3.38), ulceration (HR, 2.68), chronic sun exposure (HR, 2.31), and subsidized regimen of the health system (HR, 2.04). On the other hand, the two factors associated with greater OS were treatment with local surgery (HR, 0.27) and adjuvant treatment (HR, 0.41). The model with effect estimates (HR), 95% CIs, and the *P* value is provided in Table 4.

## DISCUSSION

According to GLOBOCAN in 2020, there were 490 deaths from melanoma in Colombia with a mortality rate of 0.8 per 100,000 people.^15^ The data reported in this research offer complementary information that had not been previously reported in our country. The finding that there were an average of 3.6 deaths per 100 patients with early melanoma per year and OS at 5 years of 83% is relevant because despite having a lower incidence in Colombia,^16^ survival is lower when compared with that reported for the North American population, where there are 5-year OS records of 99.4%.^17^ In the study of the burden of skin cancer in Colombia, the importance of some differential aspects for our country was highlighted, such as a high proportion of acral melanomas and diagnosis in more advanced stages, factors that together could modify the prognosis of Colombian patients.^16^

Unlike the histological distribution reported in other series, the number of patients with acral lentiginous type, which was documented in the present study, was remarkable. This finding had already been reported by Meijs et al^16^ and could be due to the predominance of racial heterogeneity in the population in Latin America and the fact that the Caucasian race did not predominate. This is based on the fact that in Black people and people with intermediate skin pigmentation, such as Latinos, acral lentiginous melanoma occurs especially in sites not exposed to light so that ultraviolet radiation may not be the most important etiological factor for this type of cancer in our population.

According to our results, the RFS and OS in this cohort decreases markedly between the fifth and tenth year, which leads to the clinical need to maintain follow-up of these patients for at least a decade.^18^ Factors of poor prognosis for recurrence in our patients are ECOG 3-4, stage III, presence of ulceration on histology, low income, and chronic sun exposure. By contrast, adjuvant therapy, local surgery, and Breslow <0.8 mm were shown to reduce the recurrence rate.

Concerning OS, the findings are consistent with the outcome of RFS, but with some variations finding ECOG 3-4, stage III, ulceration, low income, chronic solar exposure, and treatment with radiation therapy as factors of poor prognosis. Local surgery and adjuvant surgery were again found as protective factors.

In 2000, the ECOG index developers performed a combined analysis to establish prognostic factors in patients with melanoma, finding that an index of 1 or higher was associated with a high risk of mortality (relative risk [RR], 1.49).^19^ This has been strongly corroborated with the data from our study and makes clear the role of ECOG as the most representative independent prognostic factor for patients with early melanoma, both for RFS and OS. However, it is important to mention that this factor was not significant in the bivariate analysis, probably due to the fact of having a small number of patients at this level; however, when controlling for the other factors in the multivariate analysis, the effect as a prognostic factor was made evident.

The presence of ulceration on histology was shown to be a bad prognostic factor for RFS and OS, as other researchers have determined.^20,21^ Zhang et al established an HR of 1.96 for the presence of ulceration versus survival outcome. In this research, we have confirmed the predictive role of ulceration in our population.

Within the pathophysiology of melanoma and factors associated with its development, chronic solar exposure has been described as an important factor, especially in some types of melanoma.^23,24^ However, the value of the finding described in this investigation lies in the role this risk exposure plays as a poor prognosis factor for these patients, both for OS and RFS. It is important because it makes it possible to demonstrate the importance of prevention against this factor, which is not only associated with the occurrence of the event but is also associated with two bad prognostic outcomes in this population.

The positive effects of adjuvant treatment have been widely documented in different clinical trials.^25-27^ Our results are relevant because they allow us to corroborate them in real conditions. It is worth noting that the new adjuvant therapies (targeted or immune check point inhibitors) recently implemented in the country seem to have a promising effect. However, in this phase of the registry, there were a low number of patients who received treatment with these new drugs. Therefore, this effect should be evaluated later with a larger sample size and adequate follow-up time.

Among all prognostic factors studied, it is remarkable that surgery proved to be the variable with the greatest protective effect. This finding reaffirms the state that oncological surgery remains the mainstay of curative treatment for patients with early melanoma.^28,29^

The Breslow index has been considered a fundamental parameter affecting patients' survival.^30^ In this study, it proved to be a protective prognostic factor against the outcome of recurrence, but it was not significant in the OS models. This situation could be due to the specific weight of the other factors already described. However, since this information comes from a dynamic population registry, it will have subsequent analyses that, with larger sample sizes and longer follow-up times, may modify the described findings.

Regarding the histological type, our data show a high proportion of patients with acral lentiginous melanoma, which has been described as more frequent and a factor of poor prognosis in non-Caucasian patients.^21^ However, in the population studied, a similar distribution was found in patients with and without the events of interest.

Worldwide, different researchers have documented a higher incidence of melanoma among people of high socioeconomic status^31,32^; additionally, in countries with low-income populations, a worse prognosis has been established.^33^ This behavior seems to be consistent with Colombian population, in which a lower OS and RFS could be documented in those patients belonging to the subsidized health regimen and low incomes. This situation reflects a complex socioeconomic construct that could explain that having low socioeconomic level is a determinant for poor prognosis, both for OS and for RFS in Colombia. In a previous investigation, Sánchez-Vanegas et al^34^ had already documented that in Colombian context, there are a series of social determinants that can affect access to health services for patients with skin cancer.

According to GLOBOCAN, in a period of 5 years, it is estimated that the prevalent cases of melanoma in Colombia could be 5,268,^15^ with which we estimate that the cases reported in this investigation could be more than 10% of those diagnosed in this period of time in the country; therefore, we consider that these results are relevant for decision making at the national level.

The main limitations of this research may be due to selection bias, given by the clinical profile of the physicians who have contributed to the registry cases. Additionally, the records are susceptible to information biases that may affect the quality of the estimates. However, an emphatic search has been made to contrast the data in the primary source to give the greatest possible certainty regarding dates and results.

Despite these limitations, the data obtained provide a baseline that allows us to know the factors that have a prognostic role in patients with early malignant melanoma in Colombia and that, in addition to their clinical utility, provide information for decision making at the meso- and macro-levels of the health system.

The REMMEC registry is in continuous improvement, which will reduce measurement and information biases, broaden the base of allied institutions, and have longer follow-up times, achieving greater precision in decision making for patients with malignant melanoma in Colombia.
